# Supplementary figures and images for: Aminopeptidase MNP-1 triggers intestine protease production by activating daf-16 nuclear location to degrade pore-forming toxins in Caenorhabditis elegans
Source: PLoS Pathog. 2023 Jul 13;19(7):e1011507. doi: 10.1371/journal.ppat.1011507 (PMC10368266; doi:10.1371/journal.ppat.1011507)

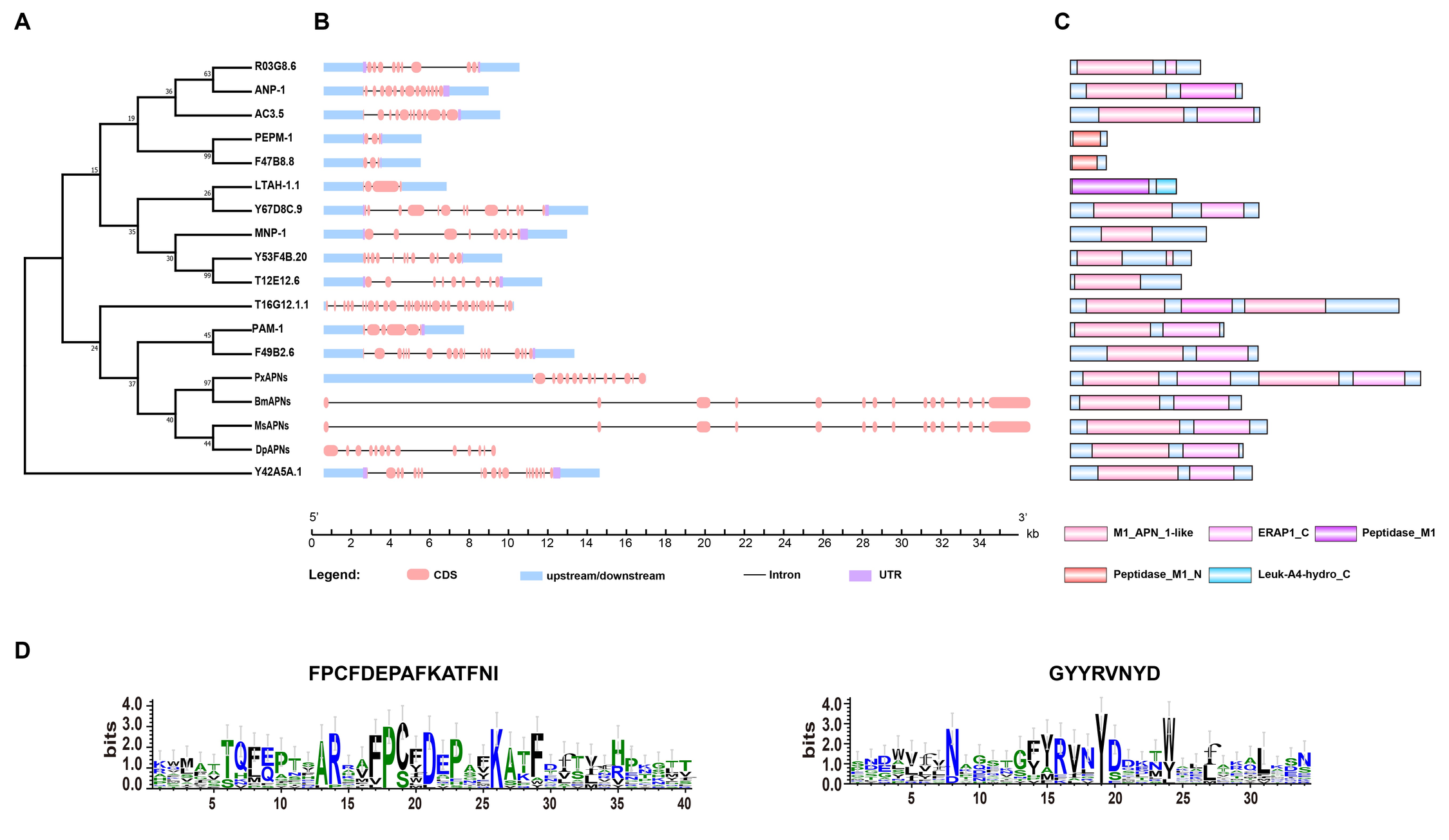

Supplement: S1 Fig — (A), phylogenetic analysis of C. elegans, Plutella xylostella, Bombyx mori, Danaus plexippus and Manduca sexta APNs. The phylogenetic tree was performed in MEGA5.1 software. (B), the exon/intron structures of the apn genes. The exons and introns were presented by red boxes and black lines, respectively. Blue boxes are upstream and downstream sequences (C), the conserved domains analysis in APNs, each domain was presented in different colored boxes as indicated in the figure. (D), WebLogo plots highlight amino acid in the FPCFDEPAFKATFNI and GYYRVNYD motifs in MNP-1 proteins. (TIF) [file ppat.1011507.s001.tif]

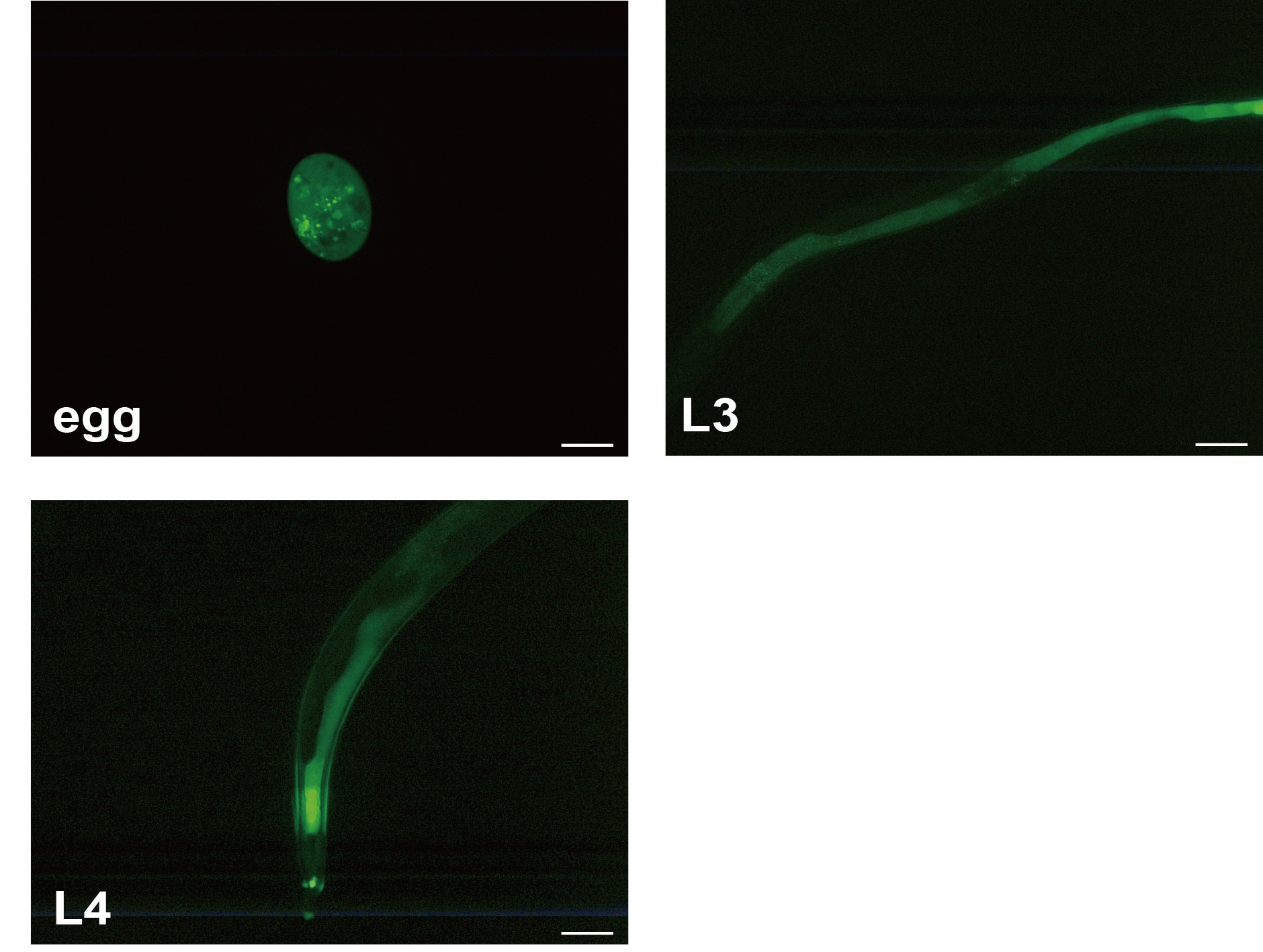

Supplement: S2 Fig — mnp-1 is expressed in the egg to adult stage. The native promoter of mnp-1 gene was generated and inserted into the pBS77, then the recombinant plasmid was injected to the gonads of mnp-1(ok2434) mutant. Transgenic worms and eggs were observed under a fluorescence microscope. The bar denotes 10 μm. (TIF) [file ppat.1011507.s002.tif]

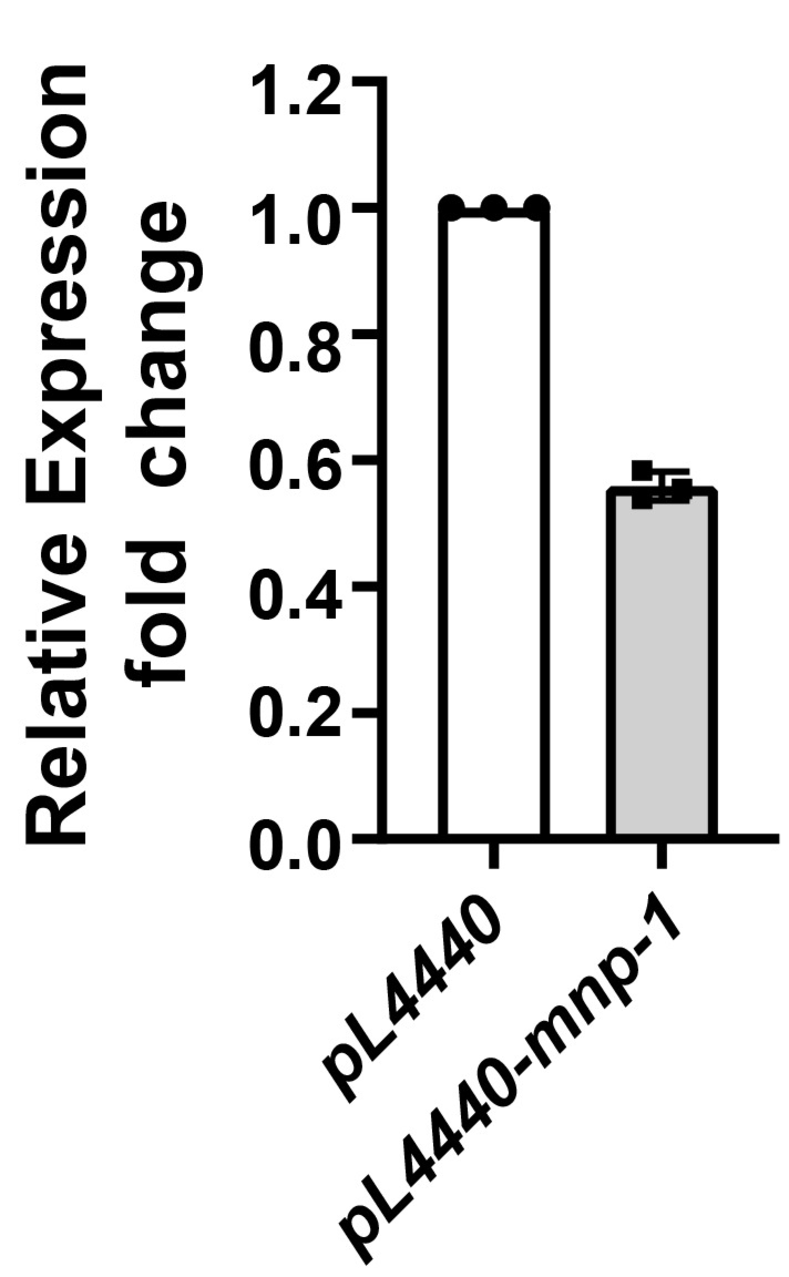

Supplement: S3 Fig — The L1 larvae of C. elegans wild-type strain (N2) were seeded on to NGM/IPTG plates with RNAi bacteria that expressed mnp-1 dsRNA until they gown up to L4 larvae. Total RNA of RNAi silenced or control worms was extracted, and the relative expression level of the mnp-1 mRNA in both samples was determined by qPCR. The mean and SD values of three independent experiments are shown. (TIF) [file ppat.1011507.s003.tif]

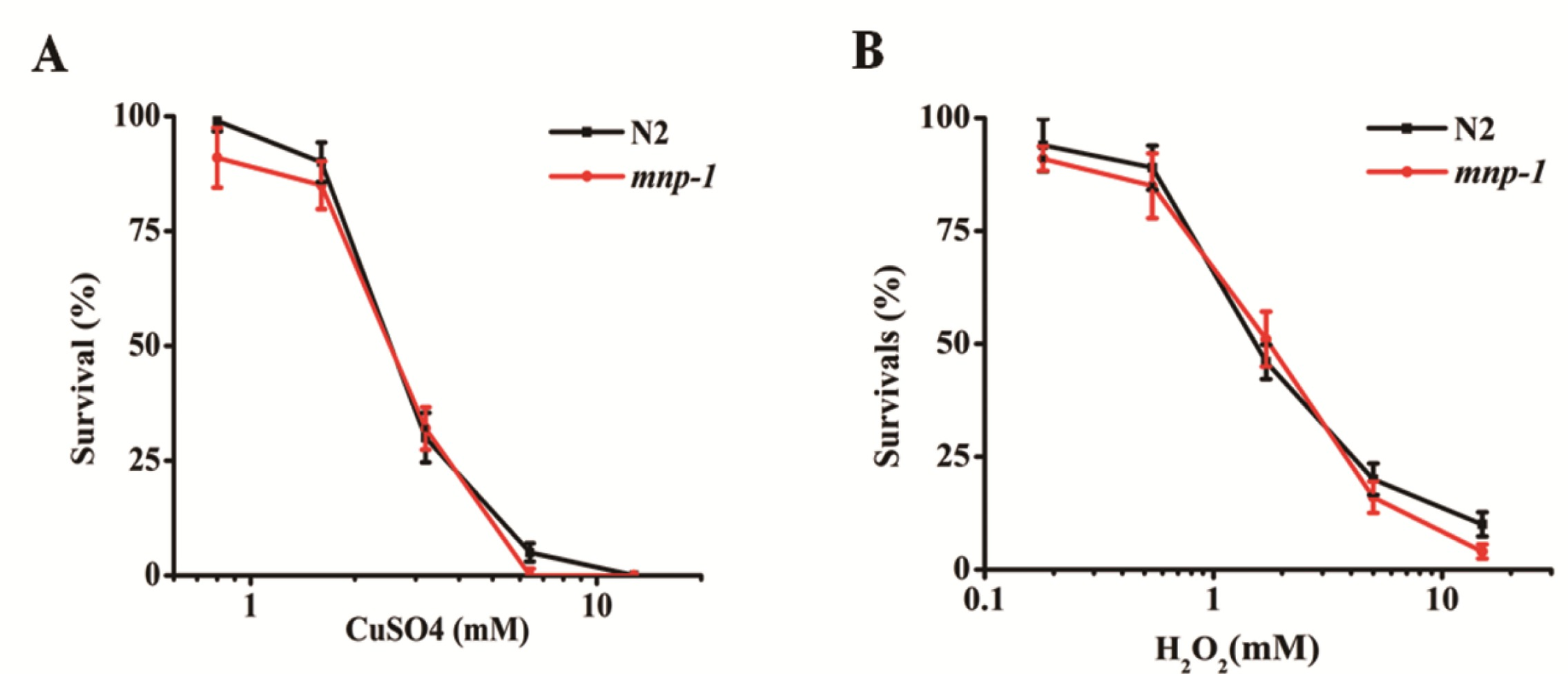

Supplement: S4 Fig — Dose-dependent mortality assay comparing sensitivity of mnp-1 mutant and wild-type N2 worms exposed to CuSO4 and H2O2. N = 3 independent experiments, containing three replication of at least 30 worms. Data points represent the mean values of three independent replicates, error bars denote the SD in A and B. (TIF) [file ppat.1011507.s004.tif]

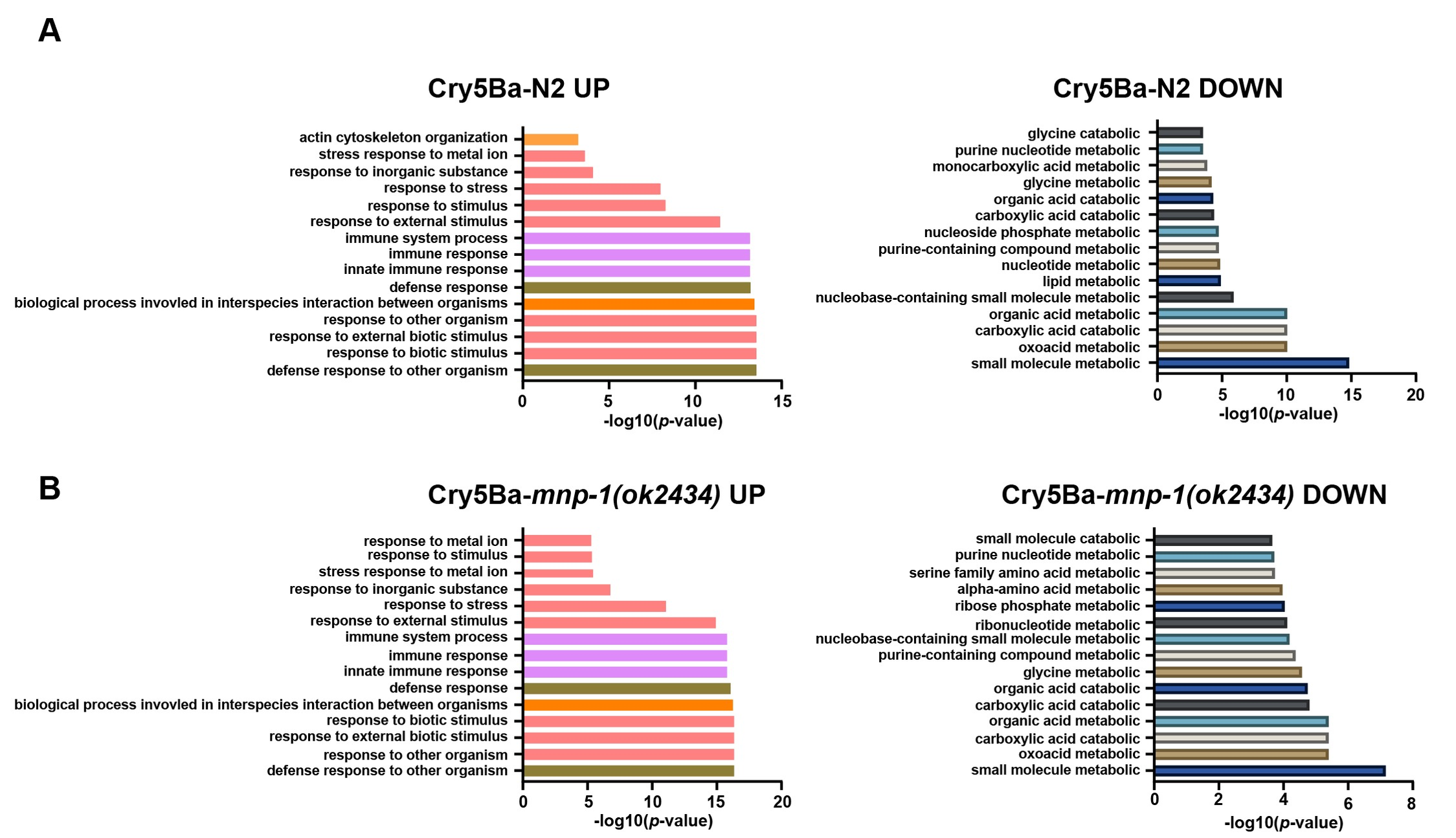

Supplement: S5 Fig — Gene ontology enrichment analysis of genes regulated by Cry5Ba in wildtype N2 (A), and mnp-1(ok2434) (B) animals. Enrichment analysis of top 15 significant upregulated or downregulated processes were shown. (TIF) [file ppat.1011507.s005.tif]

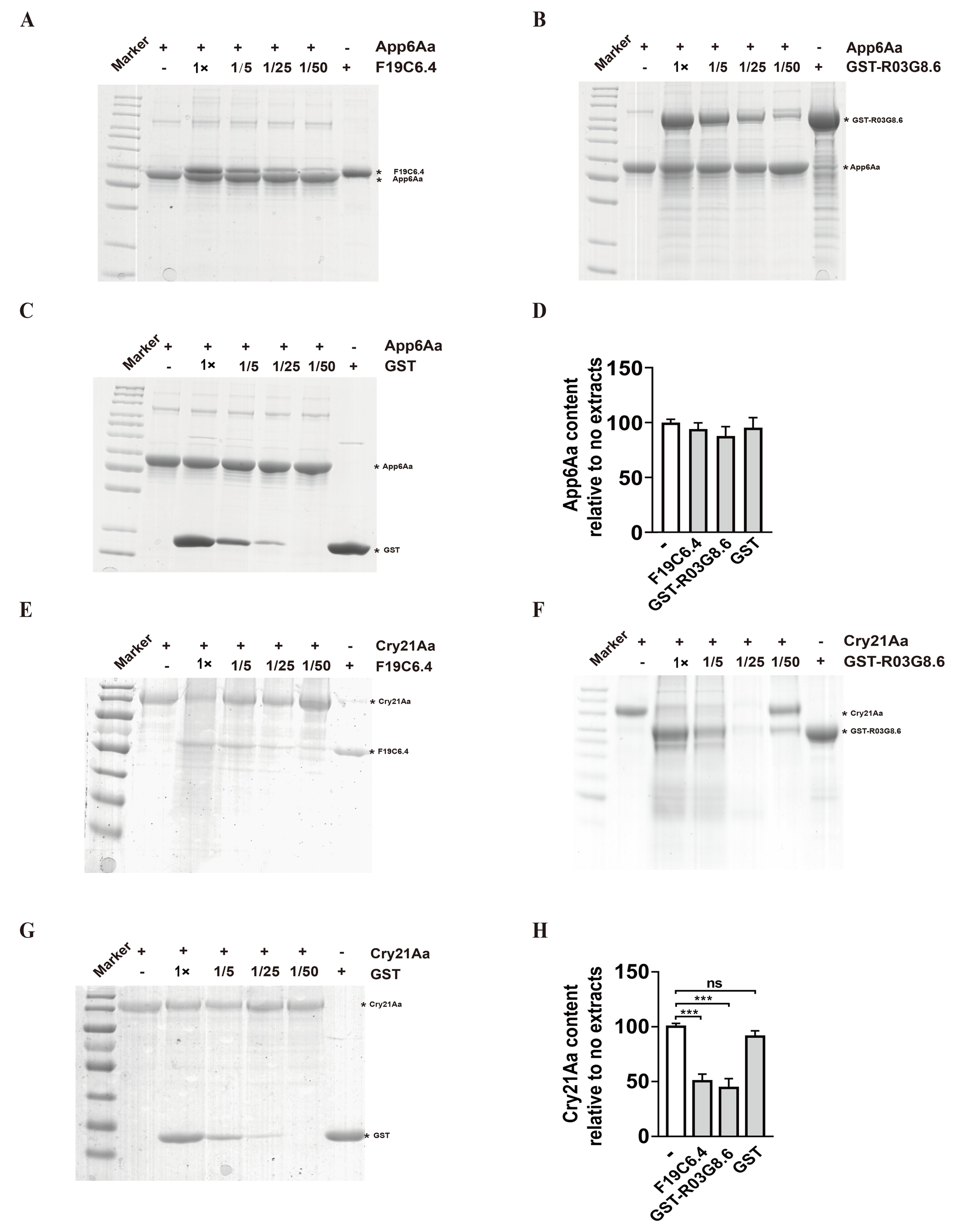

Supplement: S6 Fig — Samples of purified App6Aa or Cry21Aa were incubated with purified F19C6.4 (A and E), GST-R03G8.6 (B and F) and GST (C and G) for 4 h at 37°C, then the samples were analyzed by SDS-PAGE. The highest concentrations of F19C6.4, R03G8.6 and GST were 300 μg/ml. A negative control without extract treatment is included in the figure. (D and H), the ratio of App6Aa and Cry21Aa to the negative control after treatment with the highest concentration of purified F19C6.4, R03G8.6 and GST. Data points represent the mean values of three independent replicates, error bars denote the SD in (D and H). The p-value was determined by One-way ANOVA, ***p < 0.001 and ns indicate no significant difference. (TIF) [file ppat.1011507.s006.tif]

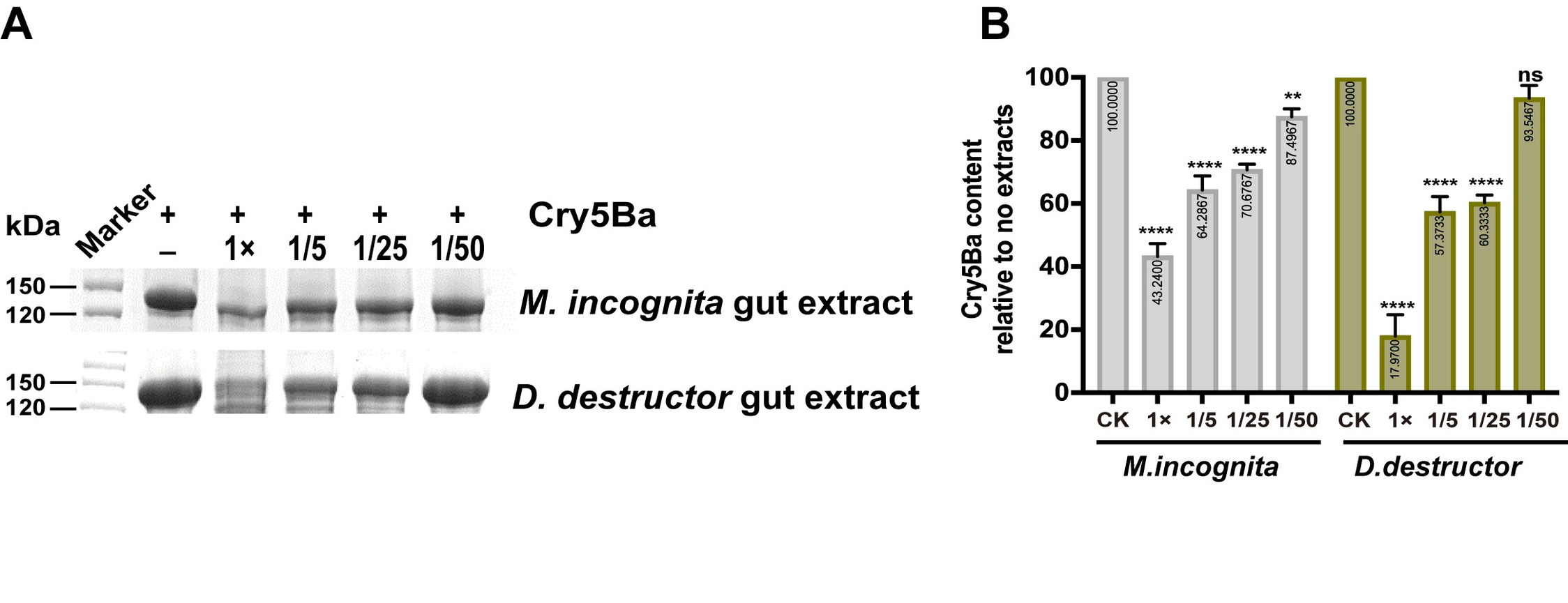

Supplement: S7 Fig — (A), crude extracts were prepared from M. incognita and D. destructor, respectively. Purified Cry5Ba was incubated with the different extracts at the same concentration for 1 h at 37°C, then the samples were analyzed by SDS-PAGE. A negative control without extract treatment is included in the figure. (B), the ratio of Cry5Ba to the negative control after treatment with the M. incognita and D. destructor gut extracts. Numbers within the column in the figure are showing the densitometry analysis of the bands. N = 3 independent experiments. Data points represent the mean values of three independent replicates, error bars denote the SD. The p-value was determined by Unpaired t-test, ****p < 0.00001, ***p < 0.001, **p < 0.01, *p < 0.05 show significant differences and ns indicate no significant difference. (TIF) [file ppat.1011507.s007.tif]
