# Supplementary material for: Aminopeptidase MNP-1 triggers intestine protease production by activating daf-16 nuclear location to degrade pore-forming toxins in Caenorhabditis elegans
Source: PLoS Pathog. 2023 Jul 13;19(7):e1011507. doi: 10.1371/journal.ppat.1011507 (PMC10368266; doi:10.1371/journal.ppat.1011507)
Supplement: Attachment — Submitted filename: Response to Reviewers.docx [file ppat.1011507.s018.docx]

**Reviewer's Responses to Questions**

Part I - Summary

Please use this section to discuss strengths/weaknesses of study, novelty/significance, general execution and scholarship.

Reviewer #1: The authors have done a nice job responding to and addressing the criticisms I raised in my initial review of the manuscript.

**Response：**We would like to express our sincere gratitude for your insightful feedback and valuable suggestions, which have been instrumental in refining and enhancing our manuscript. Your input has played a crucial role in helping us to produce an updated version, and we are truly thankful for your assistance.

Reviewer #2: This manuscript describes a new defense strategy against pore-forming toxin Cry5Ba in Caenorhabditis elegans. The authors revealed that MNP-1 up regulates two protease genes F19C6.4 and R03G8.6 through FOXO transcription factor DAF-16, which in turn leads to the degradation of Cry toxins. Overall, this is an important study and it will be of interest to scientists studying interactions between pore-forming toxins and their pathogenic hosts.

**Response：**Thank you for your comments and your constructive criticisms, as they have greatly aided us in enhancing the manuscript. Thank you for taking the time to provide us with your valuable insights.

Part II – Major Issues: Key Experiments Required for Acceptance

Please use this section to detail the key new experiments or modifications of existing experiments that should be absolutely required to validate study conclusions.

Reviewer #1: (No Response)

Reviewer #2: (No Response)

Part III – Minor Issues: Editorial and Data Presentation Modifications

Please use this section for editorial suggestions as well as relatively minor modifications of existing data that would enhance clarity.

Reviewer #1: (No Response)

Reviewer #2: I think the current version is better than before, and I have some minor comments to help further improve it:

**Response：**We sincerely appreciate your positive feedback. Additionally, thank for your comments and your constructive criticisms, which have helped us to improve the manuscript.

Minor comments:

Lines 257 and 280: The number of digits after the decimal point should be unified.

**Response：**We have revised the manuscript according to your comments, see line 260 and 283.

Lines 272-273: How to pick out “19 proteases or protease inhibitor genes” in “27 genes involved in protein degradation (Table S4)” for RNAi?

**Response:** In the first submission, we found that 27 proteases or protease inhibitors genes of the 37 immune genes are involved in *mnp-1*-mediated protection against Cry5Ba. While, in the new MS, in response to reviewers’ comments, we reanalyzed the microarray data and updated the list of up-regulated or down-regulated genes (WBGeneIDs to WormBase release WS270). We found that 27 genes are proteases or protease inhibitors, including F19C6.4 and R03G8.6 genes. However, due to changes in the worm genome version, 19 of these 27 genes were the same as those identified in the first submission. As F19C6.4 and R03G8.6 were already included in the set of proteases and protease inhibitors, we did not perform RNAi experiments on the additional 8 genes identified in the reanalysis. We will focus on the potential functions of these genes in future studies.

Line 294: R03G8.6 protein was primarily found in the nematode gonads, and the main target tissue of Cry toxins is gut. How to explain the role of R03G8.6 in degradation of Cry toxins?

**Response:** The WormBase database shows that F19C6.4 and R03G86 genes are expressed in the nematode intestine (Spencer *et al*, 2011. PMID:21177967;Han et al, 2017.PMID:28379943)(seen in line 419-423 in the manuscript), for this reason we hypothesized that F19C6.4 and R03G8.6 could be expressed in the intestine of the worms and were involved in Cry5Ba degradation in vivo. We analyzed expression of these proteases fused to GFP protein. Our data show that Cry5Ba treatment induced an increase in fluorescence in *F19C6.4p::gfp* but not in *R03G8.6p::gfp* in the gut of the nematodes(Fig 4C and 4D). We were unable to detect *R03G8.6p::gfp* expression in the gut, probably due to its low expression in this tissue. It is clear that future research will be needed to fully explain this result.

However, it is important to mention that F19C6.4 and R03G8.6 proteins degrade Cry5Ba (Fig 4G-J) *in vitro*, and that F19C6.4 and R03G8.6 mutant animals degrade less effectively the Cry5Ba protein than N2 (Fig 4E-F), supporting that both F19C6.4 and R03G8.6 proteases are involved in Cry5Ba degradation.

Lines 437-439: Other defense strategies depending on APN against pore-forming toxin in insect need in-depth discussion (see Guo et al., PLoS Pathogens, 2021, 17: e1009917 and Guo et al., Nature Communications, 2022, 13: 6024).

**Response:** Thank you for your suggestion. We have added the defense mechanism depending on APN against pore-forming toxin in the new MS. Please see in the revised manuscript in line 77-80 and 411-418.

Fig. 3 and Fig. 4: Some figures have low resolution and many letters in figures can’t be distinguished, please offer figures with high resolution; Fig. 4F is not fully displayed and the last histogram is hided.

**Response:** Thank you for pointing out our issue. Following the requirements of Plos publishing, we first checked that all figures could be displayed properly. Additionally, we have transposed the article’s Fig. 1-6 and Fig. S1-7 in PACE to ensure that all figures are presented clearly. Please refer to the manuscript’s revised Fig. 1-6 and Fig. S1-7.
