# Supplementary material for: Aminopeptidase MNP-1 triggers intestine protease production by activating daf-16 nuclear location to degrade pore-forming toxins in Caenorhabditis elegans
Source: PLoS Pathog. 2023 Jul 13;19(7):e1011507. doi: 10.1371/journal.ppat.1011507 (PMC10368266; doi:10.1371/journal.ppat.1011507)
Supplement: Attachment — Submitted filename: Response to Reviewer Comments.doc [file ppat.1011507.s017.doc]

**Please use this section to discuss strengths/weaknesses of study, novelty/significance, general execution and scholarship.**

Reviewer #1: This manuscript describes a new defense strategy against pore-forming toxin Cry5Ba in the nematode Caenorhabditis elegans. The authors demonstrated that MNP-1, a member of APN protein family, is required for C. elegans defense against Cry5Ba toxin. Conversely, in insects, APN usually act as an important receptor for Cry toxins and is necessary for the toxicity of Cry. The authors used a range of approaches to corroborate that MNP-1 upregulates two protease genes F19C6.4 and R03G8.6 through DAF-2/DAF-16 signaling pathway, which in turn leads to the degradation of Cry toxins. Overall, this is a well-organized manuscript and it will be of interest to scientists studying interactions between pore-forming toxins and their pathogenic hosts.

**Response：**Thank you for your comments and your constructive criticisms, which have helped us to improve the manuscript.

Reviewer #2: The manuscript titled "Caenorhabditis elegans aminopeptidase MNP-1 triggers intestine protease production through insulin-like signaling pathway to degrade pore-forming toxins" presents a novel way to study the function of Bacillus thuringiensis toxins.

1. The reported results revealed the classic Bt receptors, APN in C. elegans do not trigger pore-formation by Cry5Ba.

2. Microarray is an excellent way to have figured out some proteases relevant to Cry5Ba degradation.

3. It is interesting that MNP-1 (APN) is involved in insulin-like peptide signal pathway which could be further investigated.

4. RNA interference is a good way when to study the function of each individual molecule though RNAi can not completely knockdown this molecule.

5. It is good paper to call Bt researchers attention that some PFTs receptors might have opposite roles to decrease their toxicity.

**Response：**We greatly appreciate your comments and suggestions, which have been invaluable in revising and improving the manuscript. Your feedback helped us to made the updated version, and we are grateful for your assistance.

Reviewer #3: In this manuscript, Chen et al. investigate the mechanisms of host resistance to pore forming toxins using the nematode C. elegans and a crystal protein Cry5Ba produced by the bacteria Bacillus thuringiensis. The author’s genetic studies implicate a single member of aminopeptidase genes, MPN-1, in promoting resistance to Cry5Ba. Their gene expression studies implicate two protease genes under transcription control of MPN-1 in promoting resistance to Cry5Ba, which the authors propose are regulated downstream of the DAF-2 insulin signaling/ DAF-16 FOXO pathway. The characterization of proteases downstream of MPN-1 that mediate the resistance to Cry toxins is interesting and important, provided they address concerns about rigor noted below. However, the data in Fig 5 and Fig 6, and the model proposed in Fig 7, are dizzying, confusing and not substantiated by their data. The model of MNP-1/ INS-1/ DAF-2/ DAF-16 signaling is based on subtle phenotypes and a string of observations which may or may not be related. Indeed, I would be satisfied if the authors completely removed Figs 5 through 7, and focused their paper on further developing the interesting findings in Figs 1-4.

**Response：**We appreciate your comments and constructive criticisms. Your suggestions have been thoroughly considered and we have taken them in consideration to write the updated new version of the manuscript. As per your recommendation, we have removed our previous Figures 5-7. First, the results shown in Figure 6 were completely withdrawn from the manuscript. Secondly, we add new data confirming that *mnp-1* is responsible for the nuclear location of DAF-16 under Cry5Ba treatment, and that DAF-16 is able to regulate the expression of intestinal protease F19C6.4. Consequently, we have retained some of the results shown in figure 5. Finally, we have updated our model diagram that was presented in Figure 7 (now fig 6), in accordance with this new version of the manuscript. Please refer to our reply below for further details.

**Major Issues: Key Experiments Required for Acceptance**

Reviewer #1: No key new experiments are needed.

**Response:** We greatly appreciate your affirmation. In light of the valuable suggestions put forward by the other two reviewers to enhance the manuscript, we have incorporated improvements and modifications to some of the data, which can be seen in the new manuscript.

Reviewer #2: 1. At beginning, authors tested the Cry5Ba toxicity on 15-ANP mutants, nine mutants with feeding toxins and six mutant with RNAi. It would be more clear all fifteen mutants are either fed with the toxin or RNA-interfered.

**Response：**We appreciate your suggestion. During the first analysis performed to identify APN proteins involved in toxicity of Cry5Ba, we identified several *apn* genes but we were only able to get 8 nematode mutants in these proteins to perform the bioassays. The remaining *apn* genes did not have mutant nematodes available, and hence we decided to utilize RNAi to reduce their gene expression before carrying out the Cry5Ba toxicity assays. In *C. elegans*, gene function can be studied through knockout or RNAi downregulation of gene transcription. Hence, we believe that our approach was appropriate since in this study we used either the available *apn* mutants or we knocked out by RNAi the rest of *apn* genes to test Cry5Ba toxicity. Considering the above points, we do not agree that it will be absolutely necessary to silence all *apn* genes to determine the biological activity of Cry5Ba. We hope the reviewer agree with this explanation.

2. In RNA interference experiments, authors used dsRNA for experimental animals but control ones without dsRNA (line 856). The control animals should be treated with control dsRNA, such as GFP-dsRNA. Because dsRNA are delivered into the cells in endocytosis or phagocytosis.

**Response：** We performed the RNAi assay as previously reported by Kamath RS et al. *Genome Biol*. 2001. (PMID: 11178279) since this methodology was widely accepted. In these assays we utilized HT115 *E. coli* bacteria to express dsRNA. We treated the transformed HT115 cells with IPTG to induce induced expression of T7 polymerase and express dsRNA. Our control animals were treated with the same transformant bacteria but uninduced with IPTG as previously reported as a good control in these types of experiments. We have updated the manuscript, as seen in lines 503-513.

3. In Figure 3C, 3D and 3E, suppose the Cry5Ba without gut extract should be the same size, but 5Ba in 3C is smaller than in 3D & 3E. please explain or discussion.

**Response：** The protein degradation experiments conducted in this work were repeated independently three times and the images presented in the manuscript were selected from these independent triplicates. However, to better display the results, we have replaced the Fig 3C in the revised manuscript with a new figure (now in Fig 3F) to provide a clearer representation of the findings. In all these figures the Cry5Ba protein before treatment with the different gut extracts has the same size.

**Reviewer #3:**

1. Notably, the authors did not indicate in the Figure legend or the body of the manuscript how many biological replicates were performed of key phenotypic assays with the Cry proteins in Fig 1, Fig 2 and Fig 4A. In keeping with the evolving data replicate reporting requirements of most journals, and also now the NIH, I think is important for the authors to show data from at least three biological replicates for each phenotypic assay in this paper. I suggest the authors include a supplementary table with N, mean life span, p value for each individual replicate for each phenotypic assay in this paper

**Response：**Thank you very much for your suggestions. We have taken note of this issue regarding biological replicates that you raised and would like to clarify that all the data presented in the manuscript were derived from three or more independent experiments. We apologize for not explicitly stating this previously in the main text or in the figure legends. We have addressed this by including the relevant information in the figure legends of the revised manuscript.

Furthermore, we have analyzed the survival data in Fig1, Fig2, and Fig4A, and have calculated the LC_50_ value and mean lifespan upon treatment with Cry5Ba, App6Aa, and Cry21Aa, which are presented in Table 1. Moreover, we have performed statistical analysis for the *p* values for each phenotypic assay and have summarized the data in “S8 Table” that also shows N and *p* values as requested by the reviewer.

2. The authors should perform statistical tests (log rank) to compare the nematode survival data for all replicates and all phenotypic assays in this manuscript.

**Response：**Thank you for your request. We used the unpaired *t*-test in GraphPad to perform statistical analysis on the lethality and growth inhibition data of toxins in the manuscript, and calculated the differences between mutants and wild type under different treatments (as seen in line 657-667). For the lifespan assay data of N2 and *mnp-1(ok2434)* under Cry5Ba or OP50 treatment, we used Kaplan-Meier analysis in SPSS followed by the log-rank (Mantel-Cox) test (as seen in line 660-661). Regarding the analysis of the qPCR, Cry5Ba degradation ability, *daf-16* nuclear localization, and fluorescence intensity data in Figure 2, 3, 4, and 5, we used unpaired *t*-tests, one-way ANOVA, or two-way ANOVA in GraphPad to determine statistically significant differences among treatments and control groups, as shown in S8 Table.

3. Student t-test is not the appropriate statistical test for comparing multiple conditions in the qRT-PCR data throughout the manuscript. 2-way ANOVA with multiple hypothesis correction should be used instead.

**Response：** We carefully considered your suggestions and reanalyzed the qPCR data in the manuscript using the Two-way ANOVA. We also organized the results of the data comparison, which can be found in S8 Table.

4. The rescue experiment in Fig 1F should be performed with three independent lines of the extrachromosomal array to ensure the validity of the phenotypic owing to the potential for pleiotropic effects of single rescue lines. Only one line is shown. Please also indicate the promoter and the 3’UTR that was used for the rescue of mnp-1.

**Response：**When constructing the *mnp-1* rescued animals, we used the native promoter of *mnp-1* gene and 3' UTR(*unc-54*) to control the expression of *mnp-1* gene. After completing the microinjection, we selected three independent lines from the progeny for Cry5Ba toxicity assays and Cry5Ba degradation analysis. We provided a detailed description of the *mnp-1* rescued mutant construction process in the revised manuscript (as seen in lines 539-564).

5. What is the lifespan of the mnp-1 mutant on E. coli OP50 (i.e., in the absence of Cry)?

**Response:** We measured the lifespan of the *mnp-1(ok2434)* and wild-type N2 under OP50 treatment, and compared the difference between the data using Kaplan-Meier analysis in SPSS followed by the log-rank (Mantel-Cox) test. The results showed that the lifespan of *mnp-1(ok2434)* and N2 were similar under OP50 treatment, indicating that the *mnp-1* deletion does not affect the lifespan of the worms (as seen in Fig 1C).

6. For the microarray data in Fig. 3, what gene ontology categories were enriched among the genes upregulated to Cry toxin, and also those that are mnp-1 depedent? The authors could also use the tool WormCat for this analysis. The authors should perform qRT-PCR in biological replicate of a representative sample of the genes whose induction are “fully dependent” on mnp-1 to validate the microarray results.

**Response：**Thank you for your suggestion. We have reanalyzed the microarray data and the list of up-regulated or down-regulated genes was updated (WBGeneIDs to WormBase release WS270). The GO enrichment analysis identified that the upregulated genes were mainly involved in stress and immune responses, while the downregulated genes were mainly involved in metabolism (S5 Fig). We also compared the upregulated and downregulated genes after Cry5Ba treatment in N2 and *mnp-1 (ok2434)*. We found that 747 upregulated genes and 255 downregulated genes were affected by the *mnp-1* mutation. Enrichment analysis using WormCat showed that the upregulated genes were enriched in biological processes such as signaling, proteolysis proteasome, proteolysis general, and stress responses, while the downregulated genes were enriched in metabolism, proteolysis, and stress responses. We also used qPCR to compare the transcriptional differences of some upregulated genes after Cry5Ba treatment between N2 and *mnp-1 (ok2434)* strains. We found that the transcriptional level of these genes after Cry5Ba treatment was significantly lower in the mnp-1 mutant than in N2 (Fig 3 in the revised MS) supporting the fact that these genes are dependent on *mnp-1*.

7. Are DAF-16 transcriptional targets enriched in an unbiased geneset enrichment analysis of mnp-1 transcriptional targets during Cry exposure?

**Response：**Thank you for your inquiry. Based on our analysis of your Question 6, we observed that the transcriptional targets of *mnp-1* are predominantly involved in biological processes related to proteolysis proteasome, proteolysis general, and stress responses. Consequently, we proceeded to investigate the 26 genes related to proteolysis. Through information obtained from WormBase, we found that these 26 genes were regulated by *daf-2*, *daf-16*, or *daf-2/daf-16* (as shown in Table S5). These data suggest that some of these genes are transcriptional targets of *daf-16*. However, it is beyond the scope of this study to investigate whether other genes regulated by *daf-16* intersect with the transcriptional targets of *mnp-1*. We will aim to address this in our future research.

8. In Figure 3 and 4, the authors should perform three biological replicates of the experiments in Fig. 3C-G and 4E-F. Bands can be quantified for each replicate, and presented as average with error bars, and subjected to statistical testing.

**Response：**We apologize for not providing a clear description of this section's results in the manuscript. For the results in Figs. 3 and 4, we conducted three biological replicates, and the images in the figures represent one of these biological replicates. In the revised manuscript, the bands were quantified as suggested by the reviewer and we performed one-way ANOVA analysis on the three biological replicates of Cry5Ba degradation in gut extracts at four concentrations, as shown in Figs. 3G, 3I and 4F, and the corresponding statistical analysis are shown in supplemental S8 Table.

9. The authors should rescue the F18C6.4 an R03G8.6 mutants with the wildtype gene under the control of its own promoter.

**Response：**Thank you for your suggestion. We used the native promoter of F19C6.4 and R03G8.6, and the 3’UTR (*unc-54*) to constructs the rescue mutant worms (as seen in lines 539-564). Then, we selected three independent lines that were used for the Cry5Ba toxicity assays and Cry5Ba degradation analysis. Our results showed that the rescued worms of F19C6.4 and R03G8.6 were equally sensitive to Cry5Ba compared to wildtype N2. Additionally, the ability of gut extracts to degrade Cry5Ba was similar in both the rescued worms and N2. We also observed that the diffusion rate of Rhodamine-labeled Cry5Ba in the nematode intestine was similar in the recued animals to that of N2 (as seen in Fig 4). These findings suggest that the complementation of F19C6.4 and R03G8.6 can restore the wildtype nematode phenotype to Cry5Ba action.

10. The differences in bands in the Western blots in 5G and H are extremely subtle and not convincing. This is important because their model of DAF-16 regulation is based on these data, which calls into question the mechanism proposed in Figure 7.

**Response：**We appreciate you bringing this problem to our attention. Our investigation suggests that the image saturation adjustment may be the cause of this phenomenon. To enhance the clarity of our findings, we repeated the experiment and utilized Quantity One to analyze the grayscale of the bands, as shown in Fig 5H. Subsequently, we performed a significance analysis on the grayscale results. The outcomes indicated a notable decrease in the nuclear localization of *daf-16* in *mnp-1* mutants under Cry5Ba treatment compared to N2. To reflect these findings, we replaced the image and re-analyzed the grayscale, which is displayed in Fig 5.

11. The model of MNP-1/ INS-1/ DAF-2/ DAF-16 signaling is based on subtle phenotypes and a string of observations which may or may not be related. I do not think the authors have proved what they claim. I suggest removing Figs 5 through 7.

**Response:** We appreciate your suggestion. In our new manuscript, we have eliminated the findings related to INS-1 and DAF-2. Nonetheless, we have confirmed that the gut extracts of daf-16(mu86) mutant could degrade less Cry5Ba than the wildtype N2, and our findings indicate that DAF-16 promote F19C6.4 expression, suggesting that daf-16 can indeed regulate the nematode's degradation of Cry5Ba. Additionally, our latest Figure 5 demonstrates that when the mnp-1 gene is knocked down or knocked out, there is a decrease in the nuclear localization of DAF-16 upon Cry5Ba treatment. This suggests that the mnp-1 gene functions upstream of daf-16 during Cry5Ba treatment. Therefore, we retained the data on daf-16's regulation of intestinal protease expression. Additionally, we have revised the former Figure 7 into new Figure 6, which shows that Cry5Ba enters the nematode and triggers the activation of nuclear localization of DAF-16 through mnp-1 gene. This activation ultimately leads to the expression of proteases F19C6.4 and R03G8.6, which together degrade Cry5Ba in the intestine (new Fig. 6).

12. Figure 7 is an over interpretation of the authors findings and should be removed.

**Response：**We appreciate your suggestion. Based on your feedback, we have removed the former Figure 6 from the manuscript. Nevertheless, we confirmed that *mnp-1* gene regulates the nuclear location of DAF-16 upon Cry5Ba exposure, and it is locate upstream of *daf-16*. We have made revisions to our model diagram, the new model diagram (new Fig.6) illustrates that Cry5Ba treatment induces DAF-16 nuclear location through *mnp-1* gene, ultimately leading to the expression of proteases F19C6.4 and R03G8.6 that degrade Cry5Ba in the intestine.

**Minor Issues: Editorial and Data Presentation Modifications**

Please use this section for editorial suggestions as well as relatively minor modifications of existing data that would enhance clarity

Reviewer #1: I just have some minor comments to help further improve this study.

Comments:

Line 102: In the introduction, the authors should make a brief introduction of DAF-2 insulin/insulin-like growth factor-1 signaling pathway, explaining whether AGE-1, PDK-1 and AKT-1 participate in this pathway and why they are selected in this study.

**Response：**We appreciate your suggestion. However, other reviewers expressed concerns that this particular set of data did not sufficiently support our conclusions. As a result, we have made the decision to remove these data from our manuscript and thus we are not including these descriptions in the Introduction of the manuscript.

Lines 120-123: To date, the latest nomenclature and classification of insect APN family have been already elucidated (see Guo et al., Nat. Commun., 2020, 11(1): 3003). What are the differences between C. elegans and insects in nomenclature and classification of APN family? And are the differences related to the divergence of their role in Cry toxicity? I think the authors need to discuss them in the Discussion section.

**Response：**Thank you for your suggestion. We conducted an analysis of the homologous proteins of APNs in *C. elegans* and compared them to APNs from *Plutella xylostella*, *Bombyx mori*, *Danaus plexippus* and *Manduca sexta* APNs. Our findings revealed that certain APNs in *C. elegans*, including Y42A5A.1, F49B2.6, and AC3.5, contain two identical conserved domains, M1_APN_1-like and ERAP1_C. However, other APNs, such as MNP-1, only contain one of these conserved domains. This difference in sequence may contribute to APNs not serving as Cry5Ba receptors in *C. elegans*. We have provided a more detailed description of these observations in the Discussion section (as seen in lines 379-393) and we plan to investigate this further in our future research.

Line 275: Please change “In addition，” to “In addition, ”.

**Response：**We have revised the manuscript according to your comments, see line 306.

Lines 340-361: The authors attempt to demonstrate that mnp-1 functions in the upstream of DAF-2/DAF-16 signaling pathway to promote protease expression and against Cry5Ba toxin action through this part of the experiments. I think these results should move to the fifth part of results, after the results that mnp-1 could induce DAF-16 nuclear localization to activate F19C6.4 protease expression.

**Response：** Based on the recommendation of another reviewer to remove results presented in figure 6, we have accepted this suggestion and, as a result, we have not included that section in the manuscript.

Line 850: Please add a comma after “(B)”.

**Response：**We have revised the manuscript according to your comments, see line 906.

Line 922: Please leave a blank space before “p<0.01” and “ns”, respectively.

**Response：**We have carefully considered the salience callouts in the figure legends and made revisions based on your suggestions as well as those of the other reviewers. Furthermore, we have also revised the related section in response to your specific question. Please refer to line 1005-1010 for details.

Fig 7: The names of two protease genes F19C6.4 and R03G8.6 should be indicated in this figure.

**Response：**Thank you for your valuable suggestion. Based on your feedback and that of other reviewers, we have made changes to the model diagram by incorporating F19C6.4 and R03G8.6, which can now be seen in the new updated Fig 6.

**Reviewer #2: Need to check English writing, for examples:**

**Response**：We have corrected the manuscript according to your suggestion. In addition, we have asked a native English editor to revise the manuscript.

line 119: two "reported"

**Response：**Thank you for your comments, we have revised the manuscript according to your comments, see line 118.

line 137-138: simplify the sentence will be more clear "The generated transgenic mnp-1p::gfp worms express ......

**Response：**We have revised the manuscript according to your comments, see line 143-144.

line 151: . should be ,

**Response：** This sentence in line 151 (now in line 158) serves as an explanation of our intended course of action. As we have detected no grammatical errors, we have made no alterations. However, if you have marked the incorrect line number, we have sought the assistance of a native English speaker to amend any language errors and address the concern you wish to raise.

line 171: The mnp-1(ok2434) mutant and wild type N2 animals showed similar sensitive when exposed to App6Aa toxin.

**Response：**We have revised the manuscript according to your comments, see line 180-181.

line 175: remove "such as"

**Response：**We have revised the manuscript according to your comments, see line 184.

line 187-188: does NMP-1 involve in "ALL" three domain nematicidal Cry PFTs?

**Response：**In this study, we investigated the effects of Cry5Ba and Cry21Aa proteins on *C. elegans* and observed the upregulation of the *mnp-1* gene. However, we did not investigate the effects of other 3-domain Cry proteins that are toxic to nematodes. Therefore, we have revised our statement in the new manuscript to accurately reflect our findings. The revised statement is as follows: " These data indicate that mnp-1 gene was up regulated as a specific response to the exposure of C. elegans to the three domain nematicidal Cry5Ba and Cry21Aa PFTs.", as seen in line 196-198.
